# Impurities in Drug Vials Intended for Intravitreal Medication

**DOI:** 10.1155/2020/8824585

**Published:** 2020-12-02

**Authors:** Lisa Pohl, Lisa Strudel, Spyridon Dimopoulos, Focke Ziemssen

**Affiliations:** Center for Ophthalmology, Eberhard Karl University, Tuebingen, Germany

## Abstract

Sterility is an important prerequisite for minimizing the risk of severe vision loss due to endophthalmitis after intravitreal injections. We describe three cases series of incidents where an unclear contamination of the drug solution or syringe caused the injection process to stop and continue with a new preparation. During a period of 12 months with 30,502 intravitreal injections at a tertiary center, wherein 7,076 were of the drug Aflibercept drawn up from a glass vial, three cases of the critical incident reporting system relating to intravitreal injections were identified: (1) After a typical contact with the filter cannula, the glass of an Aflibercept vial was no longer intact. (2) In the course of another injection, there was a clear deposition of debris on the outer edge of the syringe when removing the attached filter cannula. (3) After inserting the syringe into the rubber top of the vial, a whitish particle of unclear origin was identified within the drug solution. Later, this contamination/particle was identified as part of the greyish rubber that was punched out with the cannula, according to the analyses of the material sent in and the manufacturer's investigations. Thus, even in busy clinics, visual inspection of the injection solution and materials used for impurities, preferably before and after pulling them out of a vial, must be an essential part of the injection process. Even when using ready-to-use prefilled syringes (PFS), vigilance must be kept high, knowing the risk of potential contamination.

## 1. Introduction

Many cases of endophthalmitis occur as clustered events, because contamination of the drug solution or pathogen sources in the environment of the intravitreal injection increases the probability of a larger number of affected eyes [1–3]. High volume clinics could potentially make the intravitreal administration of drugs more prone to errors in terms of confusion between patients and eyes but also in relation to injuries to the lens and cornea.

After possible problems such as an increase in eye pressure or toxic posterior segment syndromes due to oil bubbles remaining in the eye have been described [4, 5], the awareness of residues and contamination, e.g., of the syringe stamp, has increased again. Here, we report three different findings of our critical incident reporting system, which have occurred during a calendar year and over 30,502 intravitreal injections at a tertiary center.

## 2. Case Presentation

**Figure 1:**
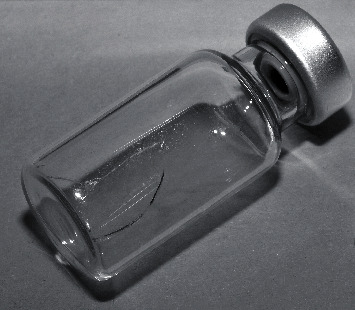
After a light and short contact with the filter cannula, fine continuous cracks on the glass vial were seen. Although no Aflibercept solution had escaped, the active ingredient—possibly no longer free of contamination—was not used.

In the first case, the injecting physician noticed fine break lines in the glass while drawing Aflibercept solution out of the vial (Figure 1). Previously, there were no issued reported during transport or preparation. The glass fragment was not dislocated; nevertheless, the sterility could not be regarded as assured. The manufacturer suspected a quality problem in the glass but stated that it had received no other reports of glass vials breaking or losing their integrity.

**Figure 2:**
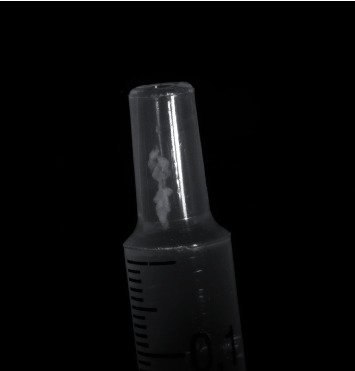
After removing the filter cannula, a kind of clot was observed that had not previously been seen on the syringe supplied.

For the second case, the attentive surgeon noticed a small contamination on the outside of the syringe tip while she was replacing the filter cannula with the injection needle (Figure 2). The suspicion arose that the particle/fibres came from the inner surface of the attached filter cannula, because it had not been noticed before. That was the reason why the drug manufacturers did not think they were responsible, as they purchased the enclosed filter cannula from a third-party supplier. Therefore, it was not further investigated what exactly the bearing material consisted of.

**Figure 3:**
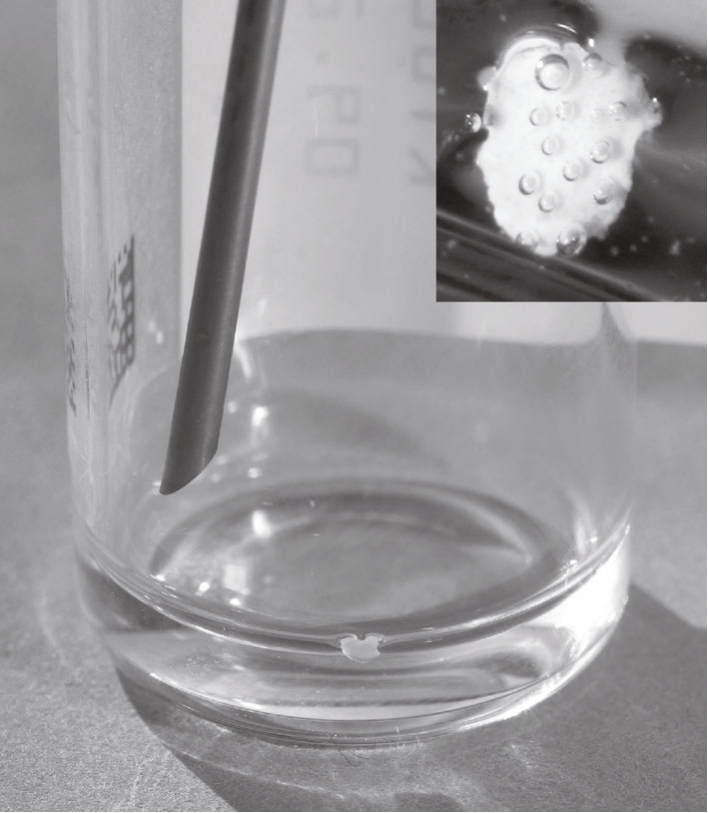
During the mounting process, the injector saw a larger particle of whitish colour (insert), so the process was aborted and the drug could not be used.

During the third injection, a whitish mass was observed which had an irregular surface structure and small air bubbles on the surface (Figure 3). The manufacturer's analysis showed that it was not a bacterial contamination but part of the greyish rubber stopper that was presumably pushed into the solution with the filter cannula when it entered (“coring”). To prevent this coring, from now on the bevel tip was inserted first and pressed downward and toward the bevel so that the bevelled tip and heel enter at the same point, when the needle is inserted into the rubber top of the vial. In addition, henceforth, an inspecting look into the glass vial was introduced regularly as part of the standardized procedures.

In all three cases, the medication of the initial vial was no longer used. In accordance with professional obligations, a notification was made to the competent authority (Federal Institute and Drugs and Medical Devices). The product was sent to the pharmaceutical manufacturer (contact: bv-complaint@bayer.com), who provided a replacement in one of the three cases.

## 3. Discussion

In this case series, three different incidents were identified as possible sources of contamination before an intravitreal injection. Intravitreal injections are indeed one of the most common procedures performed in Ophthalmology. We recommend caution during every step involved in the process. Physicians should be aware that the original solutions or their accessories may contain impurities—even in labelled drugs. These may be due to production and material defects during manufacturing or may have occurred during packaging and shipping. Even if vigilance against volatile particles and aerosols is increased during these days of the COVID-19 pandemic [6], smooth surfaces or inorganic materials may contain bacterial deposits and contamination [7, 8]. It was shown that by no means must the patients' own conjunctiva be regarded as the only source of potential contamination but also the material used [9, 10] and the oral flora of the staff [11].

In all three cases reported, standard procedure was followed in the preparation or unpacking of the vial. Although we follow and adhere to very strict aseptic rules in Germany (surgery room, overgarments for patients, eye coverage after disinfection, masks, and sterile gloves), these provide little protection against impurities or potential contamination of the drug. For the very commonly repeated procedure of an intravitreal injection, we consider it sensible and necessary not only to plan for team time-out (examination of the indication and correct eye, four-eye principle) but also to maintain an open error culture and to remain vigilant against possible contamination.

The uncertainty in the latter case was mainly due to the fact that the colour of the dislocated rubber fragment differed significantly from the grey tone above. This ambiguity could have been excluded if a careful inspection of the cuvette had become an integral part of the preparation (e.g., when unpacking the materials). We also hope that the picture presented here will facilitate an assessment and differentiation for the future. It is unlikely that material problems or contamination are limited to one drug or manufacturer only [12, 13]. A prefilled syringe can also fall down or become contaminated when unpacked; however, compared to the rate of events seen with glass vials (3/7,076≙0.4‰) during 12 months, we did not observe any such events (0/23,426) during the same period.

Although users can usually rely on the high quality of the materials used and the corresponding quality controls in the manufacturing and filling process, a high degree of vigilance should be maintained at all times. A recent review covered comprehensively the wide range of different materials used in the syringes [14]: even small measures such as tapping against the syringe can lead to an increased release of foreign impurities and silicon oil. Even if prefilled syringes (PFS) will reduce the risk of contamination during preparation and speed up the actual injection process [15], a visual inspection is still helpful: no potential sources of bacteria and thus a serious threat to vision should be overlooked. Routine and organisational efficiency must not be accompanied by a loss of the individual injector's attention, while the most frequent intervention in ophthalmology is carried out.

## Data Availability

The images of the contamination and damage supporting the conclusions of this article are included within the article. The clinical findings and images of the patients are not included, because they are irrelevant to the observed phenomena due to the interruption of the injection process (near-miss incidents).
